# Facial Muscle Activity Recognition with Reconfigurable Differential Stethoscope-Microphones

**DOI:** 10.3390/s20174904

**Published:** 2020-08-30

**Authors:** Hymalai Bello, Bo Zhou, Paul Lukowicz

**Affiliations:** 1 German Research Center for Artificial Intelligence(DFKI), 67663 Kaiserslautern, Germany; bo.zhou@dfki.de (B.Z.); paul.lukowicz@dfki.de (P.L.); 2 Department of Computer Science, University of Kaiserslautern, 67663 Kaiserslautern, Germany

**Keywords:** head mounted sensors, microphone-array, gesture recognition, wearable sensors, sound mechanomyography

## Abstract

Many human activities and states are related to the facial muscles’ actions: from the expression of emotions, stress, and non-verbal communication through health-related actions, such as coughing and sneezing to nutrition and drinking. In this work, we describe, in detail, the design and evaluation of a wearable system for facial muscle activity monitoring based on a re-configurable differential array of stethoscope-microphones. In our system, six stethoscopes are placed at locations that could easily be integrated into the frame of smart glasses. The paper describes the detailed hardware design and selection and adaptation of appropriate signal processing and machine learning methods. For the evaluation, we asked eight participants to imitate a set of facial actions, such as expressions of happiness, anger, surprise, sadness, upset, and disgust, and gestures, like kissing, winkling, sticking the tongue out, and taking a pill. An evaluation of a complete data set of 2640 events with 66% training and a 33% testing rate has been performed. Although we encountered high variability of the volunteers’ expressions, our approach shows a recall = 55%, precision = 56%, and f1-score of 54% for the user-independent scenario(9% chance-level). On a user-dependent basis, our worst result has an f1-score = 60% and best result with f1-score = 89%. Having a recall ≥60% for expressions like happiness, anger, kissing, sticking the tongue out, and neutral(Null-class).

## 1. Introduction

The face plays a crucial role in many critical human actions and interactions. Through facial expressions, we show our feelings and communicate them to others. Our faces show when we are tired, stressed, engrossed in a task, or simply lost in thoughts. Eating, drinking, speaking, and breathing, the most elementary actions of our lives, involve facial muscles. The same is valid for health-related activities, such as sneezing, coughing, snoring, or various habits, such as smoking.

With respect to human activity recognition research, there is a lot of work-related to face analysis in computer vision [1,2], but comparatively little in wearable sensing. This is because, for a long time, placing sensors on the user’s face was considered too obtrusive to be practicable (at least in wearable systems that are meant for widespread everyday use, rather than constrained lab settings). However, recently, more and more intelligent “head-mounted” devices, such as smart headphones, smart glasses, or smart hats, have become available and they have gained user acceptance. Such devices are an attractive platform for sensing face activity. Nonetheless, while facial activity affects nearly the entire face area, such devices only allow for placing sensors at particular locations (e.g., in the smart glasses frame). Consequently, sensing modalities are needed, which can infer overall facial activity from a few predefined locations. In this paper, we argue that differential body sound is a useful candidate modality. Thus, any time that our facial muscles perform an action sound is generated. While the sound by itself may be challenging to interpret, differential analysis can pinpoint the sound source, which is correlated to the muscles that have created it [3,4]. Patterns of differential sound correspond to the activation pattern of the different facial muscles (21 mimetic and masticatory muscles [5]), which are characteristic of various actions and expressions. Furthermore, differential analysis helps to mitigate noise.

### 1.1. Paper Contribution

**Figure 1:**
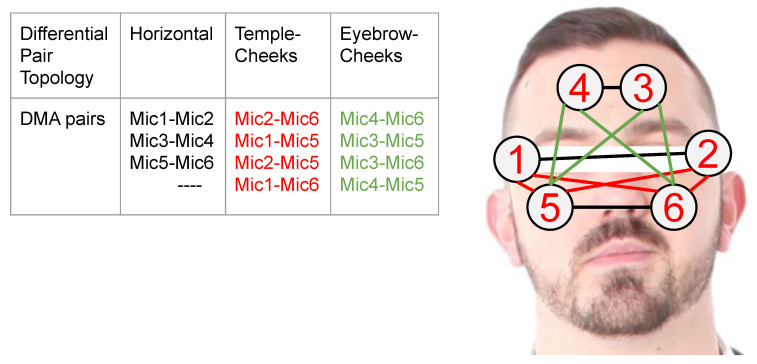
Pairs of Reconfigurable First Order Differential Stethoscope-Microphone Arrays To Detect Facial Expressions.

**Figure 2:**
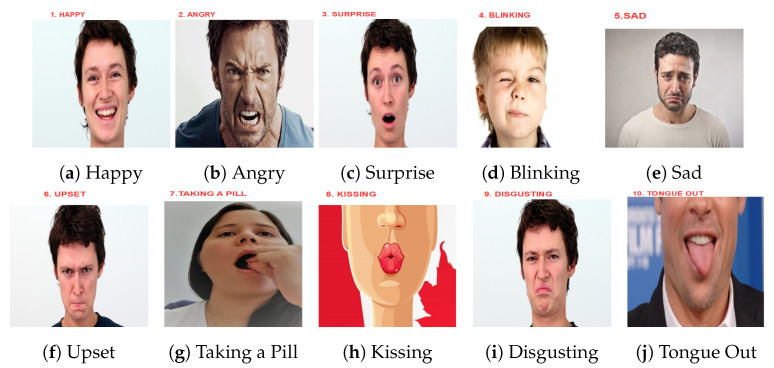
Facial Expressions/Gestures mimicking set; Happiness,upset, sadness, surprise, disgust coming from [6], angry [7] and gestures such as blinking [8], tongue out [9], kissing [10], and taking a pill.

This work explores, in detail, the potential of body sound to unobtrusively collect information about user’s facial activity and makes the following specific contributions:

1. We put forward the idea of using differential sound analysis as an unobtrusive way of acquiring information about facial muscle activity patterns and the associated facial expressions and actions.
2. We present the design and implementation of a reconfigurable signal acquisition system based on that idea. It consists of six stethoscopes at positions that are compatible with a smart glasses frame (see Figure 1).
3. We present an in-depth analysis of the system’s characteristics and the signals for various facial actions.
4. We describe the design and implementation of the entire processing pipeline needed to go from signal pre-processing to recognizing complex facial actions, including a study of the significance of different features, derived from combinations of six stethoscopes (at a set of locations that are inspired by a typical glasses frame). Additionally, the selection of best-suited ML methods.
5. We have conducted a systematic evaluation with eight users mimicking a set of 10 common facial expressions and actions (plus a NULL class of neutral face), as shown in Figure 2. Each user has recorded three sessions of 10 repetitions of each action for a total of 2640 events. Using a leave-session-out evaluation scheme across all users, we achieve an f1-score equal to 54% (9% chance-level) for those ten classes plus the non-interest class defined as “Neutral-face”. In the user-dependent case, we achieved an f1-score between 60% and 89% (9% chance-level), reflecting that not all users were equally good at mimicking specific actions.

### 1.2. Paper Structure

Section 2 provides relevant background including mechanomyography as such, general considerations related to facial expressions, and related work. Next, Section 3 describes the hardware design, its characteristics, and the calibration procedure. The experimental setup is outlined in Section 4. The data analysis, including feature extraction and classifier selection, is elaborated in Section 5, and the results are discussed in Section 6. Finally, in Section 7, we conclude our work and discuss future ideas.

## 2. Background and Related Work

### 2.1. Wearable Facial Sensing

Examples of sensors used to monitor facial activity include light, piezo-electric phenomenon [11], sound, EMG (electromyography), and one of the most recent involving TPM (textile pressure mechanomyography). A wearable light sensor solution is introduced in [12] with 17 photo-reflective sensors. The evaluation focuses on eight facial expressions(neutral, happy, disgust, angry, surprise, fear, sad, and contempt) getting results of 78.1–92.8% accuracy. The advantage of the photo-reflective idea over all others is the use of sensors that do not need contact with the skin. A disadvantage of using photosensors is the sensitivity to ambient light. To a degree, ambient light issues are also relevant to video-based facial expression detection. Facial expression recognition with piezo-film smart-glasses was proposed in 1999 by [11] and continuously developed since then [13]. To a large degree, it is complementary to the proposed sound-based approach, as it acquires mostly local information and has different sources of error.

Measuring muscular activity is usually called “myography”. Electromyography (EMG) and it is the best-known technique to measure the propagation of electrical impulses on the muscles. Gruebler in [14] and Perusquıa-Hernandez in [15] investigated EMG for facial expression monitoring. Key limitations of EMG are that the signal magnitude tends to be inversely proportional to the age; it is strongly influenced by the person’s weight and has problems with discrimination between adjacent muscles [16]. Another myography (TPM) modality is evaluated by Zhou in [17], achieving high accuracy results (82%) on classifying eyebrows’ movements and 38% for the same set of expressions as in [12] without including contempt. Unlike our approach, the TPM system is mostly limited to detecting local changes of muscle shape, and we see it as a potential candidate for fusion with our sound-based approach in future work.

### 2.2. Microphone-Stethoscope

The combination of a stethoscope’s head and a microphone is an established approach. In many cases the head is constructed using three-dimensional (3D) printed stethoscope [18] together with an Electret [19,20,21], mechanical microphones [22,23,24], or Piezo-Electrical Film [25]. Applications include automatic analysis of the cardiac, lung, and even fetal-heart rate signals [26]. Various improvements of the design have been proposed including frequency selection, noise filtering, wireless transmission and real-time feedback [19,27].

A critical issue that needs to be considered when dealing with a stethoscope is the tuning of the frequency response [28,29]. Furthermore, in [30], contact microphone, accelerometer, and stethoscope-microphone sensors were compared. The comparison was based on efficiency/signal to noise ratio for capturing heart and breathing sounds. It was found that the most efficient sensor in terms of frequency response for these specific sounds was the contact microphone. The stethoscope performed the worst of the three.

Before using the stethoscope for our experiments, we must understand how the stethoscope’s structural characteristics influence its acoustic property. In simple terms, the stethoscope is an imperfect transducer of sound for most frequencies other than the resonance frequency. Accordingly, a frequency response analysis is necessary to get to understand our electronic-stethoscope design. Changing the material of the head of the stethoscope [31] and adding/removing tubes will impact the resonance frequency [32]. Even a non-air-tight system impacts the frequency response, which is challenging to handle in a prototype. Likewise, the noise rejection depends on the construction.

In summary, the stethoscope is a challenging device to design and use. However, as will be seen in the paper, for our purpose, it significantly improves the signal-to-noise ratio (SNR) if adequately tuned.

An evaluation/comparison of stethoscopes used by nurses, a Littmann^®^ electronic stethoscope, and custom design was presented [32], where it was demonstrated that both types (electronic and passive) are resonant devices based on the experimental calculation of the frequency response using the step response analysis. A summary of this method: (1) generates a fast change in pressure on the stethoscope’s head, (2) measures the input pressure and the output signal to obtain the impulse response, (3) takes the derivative, and (4) applies the Fourier transform. In [18], a validation of a 3D printed stethoscope system was made. This time using a method called “phantom method”, which is based on a latex balloon filled with water used to simulate the skin. The balloon was stressed doing a sweep in vibration frequency to generate the response. In summary, there is a particular interest in frequency analysis and construction design as crucial parameters.

### 2.3. Differential Microphone Arrays (DMAs)

Microphone arrays, or commonly referred to as beamforming microphones, [33] are primarily designed to modify and control the directivity of the gain factor and noise robustness. Their functionality depends on the incident angle of the source [34]. Thus, the source’s position will induce changes in how the device amplifies or reduces the gain of the captured signal, implying the possibility of using them for position estimation of the sound sources [3,4].

Typically, the structural design of microphone arrays can be categorized as Additive Microphone Arrays (AMAs) and Differential Microphone Arrays (DMAs) [35,36]. There are also sub-categories that are based on the relationship shared by the intensity of the sound and the angle of incidence.

Further specifications include the microphone arrays’ configuration variations in space, denoted as first, second [37], third degrees, or higher. The structural design also varies from planar, circular [4,38,39], spherical [40], to hybrid structures [41,42].

With DMA, it is possible to tune the gain in a range of frequency or even generate a configurable resonance frequency peak. An advantage over a traditional amplifier is that there is no amplification of noise outside the selected frequencies. In the application, we use the DMA principle to focus our system on relevant sound sources inside the body (facial muscles).

### 2.4. Acoustic Mechanomyography (A-MMG)

Many activities that are performed by our body are intrinsically related to muscular contractions; therefore, activity recognition based on those contractions(myography) is an active research topic [43,44,45]. In this area, our specific interest is in the subset of mechanomyography, which involves measuring the force contraction using low-frequency sounds/vibrations (2–200 Hz) with a signal power below 50 Hz [46]. We proposed to use this method to capture the facial muscle (and to a degree tissue) movements for a specific group of gestures/facial expressions. To the best of our knowledge, there is no known research on A-MMG for the facial muscles.

The most similar to our work is [47], which investigates the replacement of electromyogram with sound myography to measure the muscle fatigue by using different microphones on the middle forearm during lifting activities, finding that Electret condenser microphone with a sampling rate of 44.1 kHz was the most stable.

In [46], sound was combined with the IMU (Inertial Measurement Unit), to monitor the muscle’s movement of patients under rehabilitation. The inspiration came from the high variability of the features of a person’s actions during a typical day, in particular patients under-recovery from a neurological injury or an accident. According to [48], MMG can also be combined with CNN (Convolutional Neural Network) for features extraction and SVM (Support Vector Machine) for regression to estimate the angle of the knee.

### 2.5. Facial Expressions

Facial expressions are linked with the state of mind and intimately connected with individual responses to external interactions. According to [49,50,51], there exist two categories for facial expressions, micro-expressions, and macro-expressions. Furthermore, the theory of universal facial expression indicates a set of facial gestures that are recognizable by the majority of the population. They form the core of the internal emotional states, independently of the cultural or geographic background, and based on the common origin of the species [49,52].

The universal core set of expressions is composed of six basic facial gestures: happiness, surprise, sadness, anger, disgust, and fear [49]. Others argue about the existence of a universal way of expressing these gestures and consider that they depend on a cultural basis [53,54]. In [55], the conclusion was that there is a strong genetic influence in the muscular movements of the face, where even a blind person shows a pattern of similarities with non-blind individuals, but differs in intensity, which implies visual learning.

Our evaluation aims to explore our hardware’s ability to distinguish different facial expressions and actions. It is motivated by the above understanding of the importance of facial expressions in order to assess users’ emotions and mental states. However, we understand that there is a big difference between users mimicking expressions and experiencing emotions. Thus, recognizing mimicked expressions is an important first step in identifying emotions, but the two are not the same.

## 3. Audio Design and Hardware

In this section, we discuss the DMA design and calibration procedure to target acoustic mechanomyography (A-MMG), when considering the frequency response of single and differential microphones. In the end, we present the apparatus employed for testing the idea of facial gesture recognition with A-MMG.

### 3.1. Differential Microphone Array Configuration

**Figure 3:**
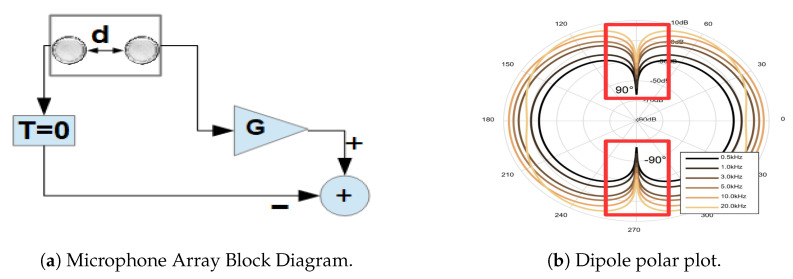
(**a**) First Order Differential Microphone Array Block Diagram. With *G* as a gain factor, *d* space between microphones and *T* time-delay between microphones. (**b**) Polar plot of first order differential microphone array(frequency vs angle of arrival). With an inter-microphone space of 16 mm, distance from the source of 50 cm, and frequencies 0.5, 1, 3, 5, 10 and 20 kHz and sound rejection at ±90 degrees(red boxes). Diagram by STMicroelectronic [35].

For this study, the DMA configuration, called first-order end-fire dipole, was used. First-order means that only two elements are subtracted from each other, as shown in Figure 3a. The subtraction works as a filter for environmental noise. End-fire connotation implies that the array will reject sound signals from sources in the ±90 degrees and enhance sound signals coming from sources at 0 and 180 degrees. This characteristic depends not only on the spatial distribution of the array, but also on the distances between each element and the geometry of the component itself. The sound wave can be simplified from a spherical-wave to plane-wave when the source is in the far-field, occurring when $r\geq 2\cdot W^{2}/\lambda$ (Fraunhofer distance) [34,56], where *W* is the largest dimension in the aperture(stethoscope-microphone head), λ is the wavelength and *r* is the distance from the opening to the source. Usually, far-field is considered to start at a distance of two wavelengths away from the sound source.

Moreover, the dipole term describes the form of the polar graph of intensity vs. angle of arrival for different frequencies, as depicted in Figure 3b. The diagram in that figure assumes: an inter-element space of 16 mm, a distance from the source of 50 cm, and frequencies of 0.5, 1, 3, 5, 10, and 20 kHz. In our case, the wave simplification is not valid, when considering our sound sources as the face muscles’ surface.

The End-fire design is shown in Figure 3b, where *G* is the gain factor difference of the microphones, *d* is the inter-spacing, *T* is the time delay between the two microphones. We will assume T=0, but this value can also be configured according to the formula T=D/V, where V=343 m/s (sound velocity). This adjustment will change the polar-plot (directivity pattern) in Figure 3b and dipole will change to cardioid (heart shape) representation [35]. This directivity pattern depends on the number of elements inside the array, the inter-spacing, length of the aperture (Area of sensing), and their differences in the frequency response [33,34,35].

### 3.2. Calibration Procedure

The frequency response and gain of the stethoscope microphone is highly sensitive to the mechanical design parameters, which are not precisely identical across the individual devices. Furthermore, in the DMA configuration, the spatial separation of the microphone is an additional influence. Thus, before our sensor configuration can be used for facial activity recognition, a calibration step is needed. This includes gain differences for each of the individual microphone as well as the first-order differential case.

#### 3.2.1. Single Microphones Discrete Frequency Response Calibration

**Figure 4:**
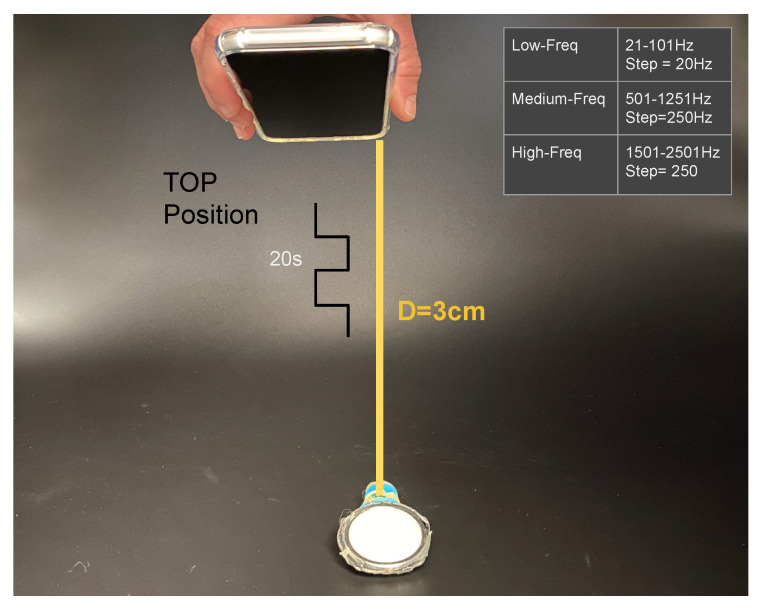
Calibration experiment one, showing sound-source (phone) and stethoscope-head at 3 cm separation. Frequency range of the sound square wave with 20 s duration at each frequency.

We expose each stethoscope-microphone individually to a range of frequencies in the low-spectrum (21, 41, 61, 81, 101 Hz), in the middle-spectrum (501, 751, 1001, 1251 Hz), and in the high-spectrum (1501, 1751, 2001, 2251, 2501 Hz) for 20 s each frequency with a square-wave signal. We used an android application in a Samsung Galaxy S8 on top of the stethoscope-head (3 cm separation) at the highest available volume to generate the signals (Figure 4). The experiment was conducted in a quiet room with only a single person present.

**Figure 5:**
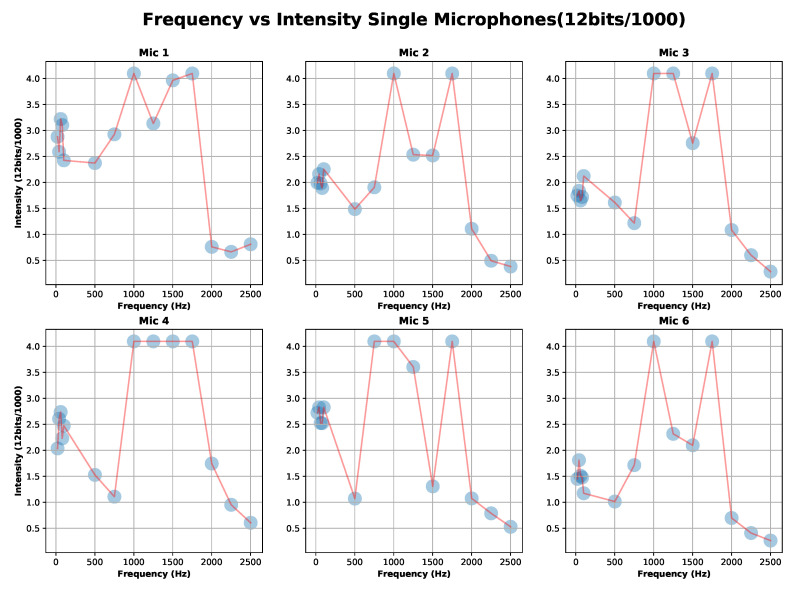
Calibration experiment one results: capture signal (ADC coded 12 bits) for each microphone-stethoscope for “Area1” = Low-freq (21–751 Hz), Area2 = Middle-freq (1001–1751) and Area3 = High-freq (2001–2501).

The calibration results of the six microphones are shown in Figure 5. It can be seen that the peak to peak value of the signal (ADC value) is not flat for the entire frequency range. Specifically we can distinguish three significant areas; “Area1” = Low-freq (21–751 Hz), “Area2” = Middle-freq (1001–1751), and “Area3” = High-freq (2001–2501).

Let us define A=V·G, where *G* is the gain of the microphone and *V* is the response of the microphone to a sound input at G=1. We can see that A=V·G depends on the frequency-area and the stethoscope-microphone used. This result was to be expected due to a prototype design developed by hand.

According to [46], the relevant frequency range is within “Area1”. The corresponding values of the minimum, maximum, and mean for each microphone are shown in Table 1. The selection of the microphones’ gain in “Area1” will be discussed in more detail in Section 6. Based on the values that are presented in Table 1 and assuming ideal conditions, we estimate the gain differential maximum error in the case of Mic5max−Mic6min to be of 4.03. Accordingly, the gain discrepancy of our microphones could be a factor of 4 in “Area 1”.

The detection in “Area2” could be improved by merely using an ADC with more resolution as used in [47] (32 bits). For “Area3”, the signal has a lower gain in the complete response.

**Figure 6:**
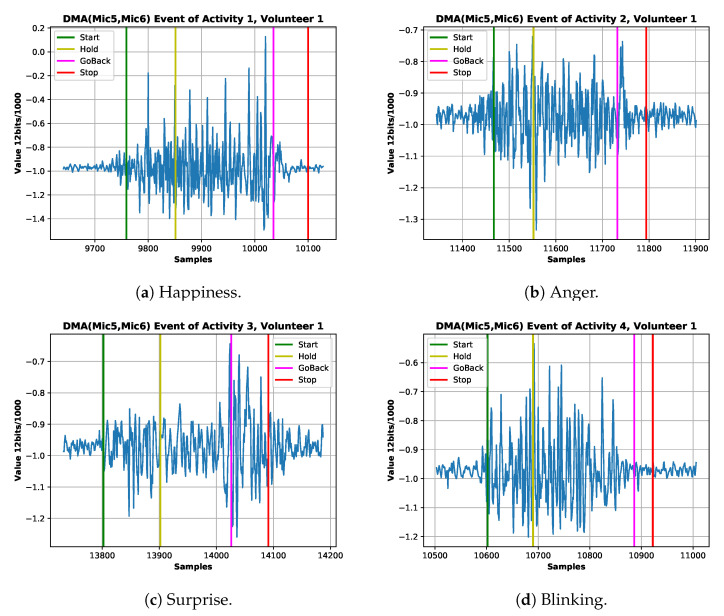
Example ADC coded signal of four expressions from the best volunteer. (**a**) Happiness signal of the first session signaling start,holding,go-back and stopping gesture. (**b**) Anger signal of the first session signaling start, holding, go-back and stopping gesture. (**c**) Surprise signal of the first session signaling start, holding, go-back and stopping gesture. (**d**) Blinking signal of the first session signaling start, holding, go-back and stopping gesture.

In summary, the sound signal of interest is defined by a frequency range between 2 Hz to 200 Hz (50 Hz Bandwidth) [46] and non-drift time series, as shown in Figure 6. Additionally, a sound signal can be considered stationary (constant mean and standard deviation) when the time window is of few milliseconds, as established in [57], which is not the case because our sound signal window is around 2–3 s duration. The non-stationary condition can also be seen in the Figure 6 according to [58].

Next, we apply the 1st order DMA (Differential microphone array) with a matching factor $MF=\frac{A_{y}}{A_{x}}$ before the subtraction and test the influence of the gain. Let us assume that $A_{x}$ is the ADC value of microphone 1 and $A_{y}$ is the ADC value of microphone 2 from the calibration (Table 1).

The matching factor MF is used in Equation (1) to map the output of microphone 1 to the output of microphone 2, resulting in Equation (3).

- DMA 1st order with matching factor is called $cal_{dma}$:
  (1) $$\begin{array}{c} cal_{dma}=\frac{A_{y}}{A_{x}}\cdot M_{x}-M_{y}\;,\mathrm{where}\;A=V\cdot G\;\mathrm{and}\;M\;\mathrm{is}\;\mathrm{the}\;\mathrm{ADC}\;\mathrm{value}\;\mathrm{of}\;\mathrm{the}\;\mathrm{microphone}. \end{array}$$
- By substitution of A=V·G in $cal_{dma}$:
  (2) $$cal_{dma}=\frac{V_{y}\cdot G_{y}}{V_{x}\cdot G_{x}}\cdot M_{x}-M_{y}$$
- Assuming same sound source position and geometry, only gain discrepancies.
  $$V_{y}=V_{x}$$
- By substitution of $V_{y}=V_{x}$ in $cal_{dma}$.
  (3) $$cal_{dma}=\frac{G_{y}}{G_{x}}\cdot M_{x}-M_{y}$$

#### 3.2.2. Differential Microphones Discrete Frequency Response Calibration

**Figure 7:**
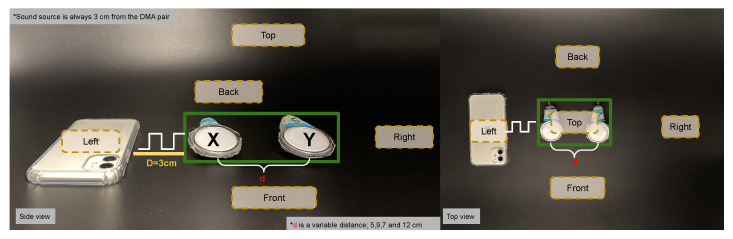
Calibration showing sound-source (phone) at 3 cm from DMA; microphone “X” and microphone “Y” with a variable inter-microphone distance “d” (5, 7, 9 and 12 cm). Frequency range of the sound square wave with 20 s duration at each frequency and for each source position (back, right, left, top and front).

The second calibration step concerns a set of two stethoscope-microphones treated as a DMA(Differential Microphone Arrays) of the first-order end-fire dipole, as shown in Figure 3a. We must remember that in a DMA, the separation between the elements of the array alters the gain frequency pattern (see Section 2). We conducted a discrete frequency sweep for four distances between the microphones-stethoscope pair (5, 7, 9, and 12 cm). The distances were the average values of separation between the six stethoscope-heads placed on the volunteers’ faces. Also, due to the dependency on the source position and the gain factor, the sound source was placed in five different locations; top, back, front, right, and left, as shown in Figure 7, at 3 cm fixed distance from the system. One repetition of the experiment per position (back, right, left, top, and front) was done. The setup is shown in Figure 7.

We focus on first-order DMA combining pairs of stethoscope-microphone, as shown in Figure 1. We consider 11 pairs in three categories; DMAs-Horizontal: including DMAs from $Temple_{left}-Temple_{right}$, $Eyebrow_{left}-Eyebrow_{right}$ and $Cheek_{left}-Cheek_{right}$ and the self explanatory temples-cheeks and eyebrows-cheeks cases. With respect to the position dependency of the sound source, we selected the third Horizontal DMA pair from Figure 1 to obtain the intensity value ADC coded as $A_{x}-A_{y}$ for five positions depicted in Figure 7. We were always assuming a negligible difference in the stethoscope-microphone geometry.

**Figure 8:**
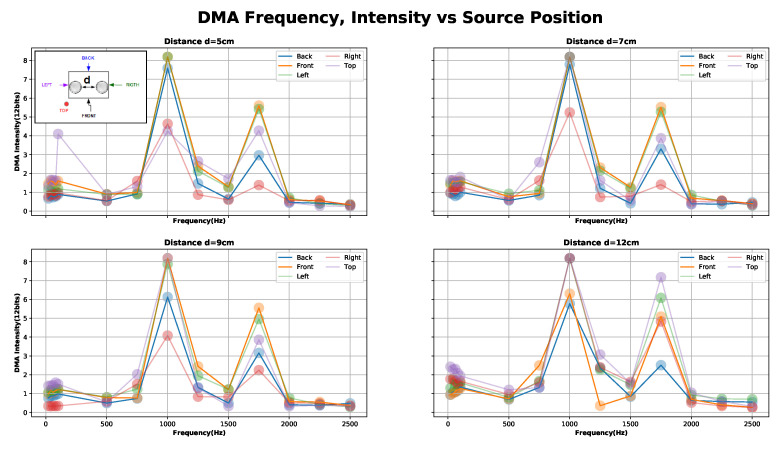
Calibration experiment two results: capture signal (ADC coded 12 bits) in a first order Differential Microphone Array (DMA) configuration between source positions (top, back, front, right and left) for “Area1” = Low-freq (21–751 Hz), Area2 = Middle-freq (1001–1751) and Area3 = High-freq (2001–2501).

In Figure 8, we have shown that the signal’s gain of our system does not only depend on the frequency range, but also source placement. We can likewise observe that in “Area1” with the source at the TOP position, the gain is slightly higher than in the other settings. Besides for 9, 7, and 12 cm inter-spacing (distances between microphone’s heads), the response in “Area1” does not show a resonance peak. This is in line with the formula $F_{null}=V/2\cdot D$, where V=343 m/s (sound speed), D=inter−spacing and the $F_{null}$ is the tuned frequency [35]. In the case of DMA, end-fire dipole implies the need for $D=V/2\cdot f_{null}$.

In our setup, we could not achieve tuned directivity in the low-frequency range for reasons of geometry. The minimum distance of our design (5 cm) has a tuning frequency of $f_{tune5cm}$ = 3.4 kHz while the maximum (12 cm) has $f_{tune12cm}$ = 1.4 kHz. At the same time, our stethoscope-microphones amplification region is 1.4 kHz $\leq f_{tune}\leq$ 3.4 kHz (assuming negligible stethoscope’s head area and equal gain).

Overall, the choice of DMA configuration is motivated by two considerations. First, DMA and AMA(additive microphone array) are the most straightforward configurations of microphone-arrays. Secondly, DMA allows noise resilience to common environmental sound interference(subtraction). With AMA, this would increase(addition), and we would need an additional technique for environmental sound removal. Third, we would like to make our first step in facial gesture recognition before going into a more complicated array design, for example, the first order DMA end-fire cardioid. In an end-fire cardioid the delay *T* in Figure 3a will no longer be zero, and follows the T=D/V equation, which in term of the sampling time ($T_{s}$) is $T=T_{s}\cdot N$, with (*N*) being the number of delays added to one of the microphones. In theory, the highest captured frequency would be around $f_{capture}\leq f_{null}/2$ for the signal coming from sources parallel to the array plane at zero degrees(microphone without delay). Nevertheless, to achieve higher gain at low frequency with our maximal distance between stethoscope-microphones head at 12 cm, the delay would have to be implemented by a fractional delay filter. Alternatively we would have to increase the sampling rate to 2.86 kHz, resulting in $f_{null}$ = 1.43 kHz and the highest $f_{capture}\leq$ 714 Hz. Additionally, in the case of not matching the delay with the distance *D*, we could get into sub-cardioid(delay larger) or hyper-cardioid(delay smaller) [39], plus many other configurations with multiple microphones, as mentioned in Section 2.

In conclusion, we are using Equation (3) as a calibrated first order DMA(Differential array) for the 11 combinations to reduce gain discrepancies. Assuming negligible difference in the geometry of the microphone-stethoscopes and focusing on the “Area1” = Low-freq (21–751 Hz) of the Figure 8 with sound source on the surface of the stethoscope (TOP case). The system remains with only the space position dependency. Therefore, in the presence of a common sound source, the ADC coded value will capture the space position relevance for such sound.

### 3.3. System Architecture and Implementation

**Figure 9:**
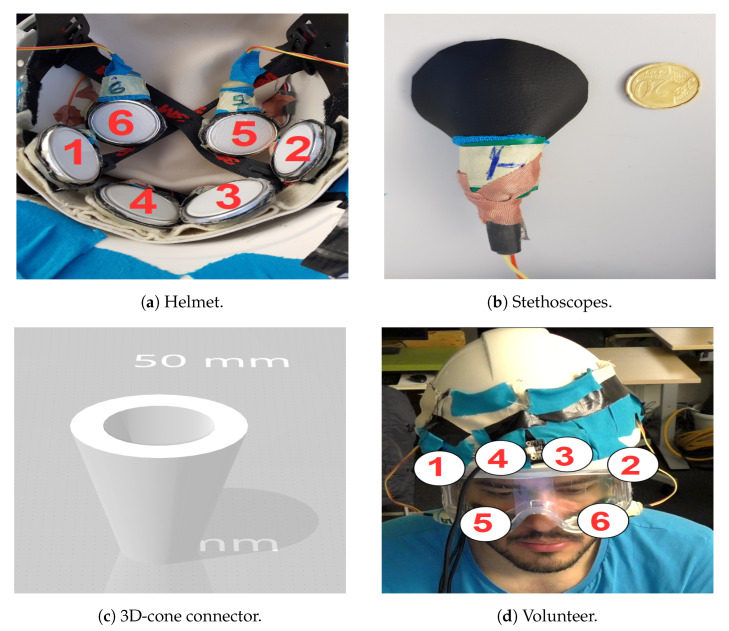
(**a**) Helmet inside, four fixed stethoscope-microphone in elastic-band and two loose. (**b**) Back side of stethoscope head with leather cover and size comparison. (**c**) Three-dimensional (3D) cone connector between Electret microphone and nurse stethoscope head. (**d**) Stethoscope-microphone on the face of volunteer using construction helmet and goggles.

The prototype needs to be wearable and suitable for volunteers with different head/face sizes to facilitate the planned experiments. Figure 9 showsthe design we have chosen. It consists of six stethoscope-microphones placed inside a construction helmet in Figure 9a, four of them fixed into an elastic band, to fix them around the temples and the eyebrows of the subject. The other two are attached the cheeks using construction goggles, as shown in Figure 9d. These particular positions were selected to match a typical glasses’ frame with the future goal of a ubiquitous design.

We have designed a three-dimensional (3D) cone in Figure 9c to connect the microphone to the stethoscope-head in Figure 9b, so we could guarantee as much as possible an air-tight design. The stethoscope-head is covered with a leather-like textile to reduce outside noise further.

The electronic components selected were six Electret Microphone boards attached to low power, low-cost pre-amplifier (MAX446) with adjustable gain (from Adafruit [59]). We have decided to adopt this type of microphones to facilitate fast prototyping as it has a built-in amplification circuit and is easy to program. For micro-controller, we have chosen an Adafruit Industries ESP32Huzzah development board [60]. With two cores running at 240 Mhz and 2 ADC (Analog to digital converter) with 12 bits resolution, a signal to quantization noise ratio (SQNR) ≈ 72 dB and a DC-Bias of VCC/2 at Vin = 3.3 V with a precision of 0.805 mV. All of the above make it suitable for collecting fast data coming from the analog microphones. In addition, it has Bluetooth low energy (BLE) Bluetooth serial and Wifi for easy communication.

The sampling rate of each microphone was set to 200 Hz. Although the hardware could handle up to 3 kHz, a lower sampling rate has several advantages. First, noise increases with a high sampling rate and we would require more complicated noise rejection techniques. Second, the signal of interest is localized in the low-frequency (lower than 200 Hz), as mentioned in Section 2.4. The data transmission protocol to the PC was by UART (Universal asynchronous receiver-transmitter) at 1 Mhz for robust data collection. The data was collected using a user interface developed in python 3.6.

**Figure 10:**
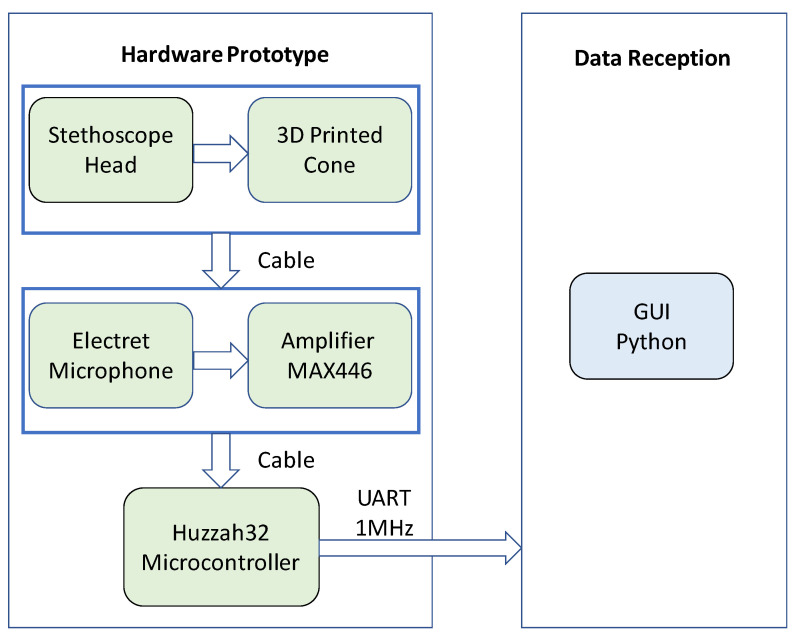
Apparatus block diagram.

The decisions were made to construct a wearable prototype to evaluate the feasibility of using the stethoscope-microphone sensors to detect gesture/facial expressions. Thus, it was kept in mind that the design was not to be an end-product. A block diagram of the system can be seen in Figure 10.

## 4. Detecting Expression Experiment Design

### 4.1. Facial Expressions

We have conducted an experiment where volunteers were asked to mimic a set of facial actions shown to them on generic pictures in order to validate our approach. The actions were selected to imitate basic human facial expressions, like happiness, anger, upset, sadness, surprise, disgust, and gestures, such as blinking, sticking the tongue out, kissing, and taking a pill (10 activities), as shown in Figure 2. This includes five (fear not included) out of six of the core expressions (Section 2.5). According to the Warsaw study [6], fear is often confused with surprise, even by human observers. A possible explanation is that when we are frightened, we are also surprised. Instead, we added three flirting gestures (blink, kiss, and tongue out). Finally, taking a pill is a highly relevant gesture from a practical point of view (medication monitoring). In Figure 2, happy, surprise, sadness, upset, and disgust are pictures from the Warsaw photo set [6] and from public domain the faces angry [7], blinking [8], kissing [10], and tongue-out [9].

### 4.2. Participants

The participants were five women and three men around the ages of 24–29. They come from countries like Venezuela, Brazil, and India, all of them were students at the Technical University of Kaiserslautern (TU-KL), Germany. This provides for reasonable and ethnic diversity. To the best of our knowledge, all of the volunteers had normal eyesight or and could perceive the presented expressions without prescription glasses. The participants did not have a problem identifying the facial expression on themselves and others. All of them signed an agreement following the policies of the university’s Committee (Technical University of Kaiserslautern) for the protection of human subjects, which approves experimental protocols at the university. The experiment was video recorded for further private analysis. There were no reported pandemic or any contagious disease outbreak in the region during the experiment recording time.

### 4.3. Experiment Procedure

We followed the protocol described in [54], asking the volunteers to emulate the facial actions with as little variability as possible. In addition to the pictures, the name of the expression was provided. While this clarified the action for some participants, it was, however, perceived as confusing by others. The experiments were performed in a closed office with a carpeted floor and only two persons inside, the person monitoring the experiment and the participant.

The hardware was fixed on each volunteer’s head with all six microphone-stethoscopes at the same time. The set of facial expressions was displayed in random order with ten repetitions per activity for eight volunteers. We used color-coded lights to prompt the subjects to start and stop mimicking each repetition of each action. Accordingly, when the graphical user interface (GUI) showed green light, then the participant had to start making the respective action, and he/she stopped and went back to neutral expression when the red light went on. The duration per action was between two to three seconds, including the rest time (Neutral event). This neutral event was considered as the null-class, because it is trained with data outside our gesture dictionary.

The same experiment was repeated three times (sessions) per volunteer with a gap/resting period of a few hours or days. The gap is introduced to ensure the participants’ facial muscles are properly rested, as the mechanomyography could be used as a measure of the fatigue of muscle [47,61]. We are using the definition of fatigue as “any reduction in the force-generating capacity, regardless of the task performed” [62]. We collected 240 samples per activity (10 repetitions per gesture, three-session for eight volunteers) for a total of 2640 (Null Class included) samples. As an example of the captured data, the state machine of the signals of four expressions from the first volunteer is in Figure 6.

## 5. Data Analysis

In this section, we explain our data analysis approach, including features calculation, feature selection, and classifier selection. We evaluate our approach on an individual basis (user-dependent) and the dataset as a whole (user-independent). All of the validation is carried out with the leave-session-out scheme.

### 5.1. Feature Extraction

We first used the pyAudioAnalysis (An Open-Source Python Library for Audio Signal Analysis) [63], to investigate an initial set of features, such as; zero-crossing rate, energy, the entropy of energy, spectral (centroid, spread, entropy, flux), roll-off, Mel frequency cepstral coefficients, chroma vector, and chroma deviation, and has fast plotting capabilities. For a more detailed analysis, we then switched to Tsfresh (Time Series Feature Extraction based on Scalable Hypothesis tests) version 0.16.0 [64] also made for python and MIT license.

We used the Tsfresh library to extract 754-time features per input of DMA (11 in total), having a total of 8294 features. For feature selection, Tsfresh provides a feature extractor based on the vector of *p*-values, where smaller the *p*-value means a higher probability of rejecting the null hypothesis. To select the threshold for the *p*-value, the library uses the Benjamini-Yekutieli (BY) procedure [65]. A summary of the BY procedure would be: (1) organize the *p*-values from lower to higher (step-up) and (2) select a small group of them, where the boundary between the selected features is set by the condition $P_{\left(k\right)}\leq \frac{k}{m\cdot c(m)}\alpha$; where $P_{k}$ is the *p*-value, *k* is the last *p*-value to be declared as valid for a given α (rejecting the null hypotheses), *m* is the total number of hypothesis/features and c(m) is a constant defined as c(m)=1 when the features are independent or positively correlated, and as $c\left(m\right)=\sum_{i=1}^{m}\frac{1}{i}$ when there is an arbitrary dependency (selected case). This relationship is a simple graph of *p*-values as dependent variable (“y”) and independent variable (“x”) equal to the range of 1...k, with slope = $\frac{\alpha }{m\cdot c(m)}$.

The signals from the different microphone array combinations in the Figure 1 were used as input to the feature extractor (Tsfresh) follow by standardization (mean=0, unit-variance) and a feature reduction. The reduction of the features was done using the Benjamini-Yekutieli technique per volunteer and then selecting the top commons sixteen features presented in the list below, then these sixteen features were feed to a second round of extraction by each DMA (11 in total), given a maximum number of extracted features equal to DMAs×16=176. These sixteen were extracted for both cases; user-dependent and user-independent tests.

The sixteen retained features are:

- F1 80% quantile
- F2 10% quantile
- F3 Absolute FFT coefficient #94
- F4 Absolute FFT coefficient #38
- F5 Absolute FFT coefficient #20
- F6 *p*-Value of Linear Trend
- F7 Standard-Error of Linear Trend
- F8 Energy ratio by chunks (num-segments = 10, segment-focus = 1)
- F9 Energy ratio by chunks (num-segments = 10, segment-focus = 8)
- F10 Autocorrelation of lag = 2
- F11 c3 = $\left\{E\right\}[L^{2}{\left(X\right)}^{2}\cdot L\left(X\right)\cdot X]$ lag = 3
- F12 Count below mean
- F13 Minimum R-Value of Linear Trend (chunk-length = 10)
- F14 Largest fixed point of dynamics (PolyOrder = 3, #quantile = 30)
- F15 Ratio beyond r-sigma (r = 1.5)
- F16 Mean change quantiles with absolute difference (qH = 1.0, qL = 0.0)

Four of the most relevant features in the list above are connected with the quantile definition (features F1, F2, and F16). Quantile is the value below which a defined percentage of the data is expected to lie. For example, the first row of features list implies, a crucial feature of our data-set is a distinct value limiting the 80% of the data to be below it, in simple words, an upper threshold. In second place in the number of appearances, we found the FFT (Fast Fourier Transform) and linear least-squares regression (Linear Trend, features F6, F7, and F13). Here, the linear regression that was assumed the signal to be uniformly sampled (true for our case). Inside the linear trend characteristics, our focus is in *p*-value with the null hypothesis = “the slope equal to zero”, correlation coefficient (*r*-value), and the standard error of the estimation (stderr).

Next, is the energy ratio by chunks (F8 and F9). The procedure to extract this from our signal is; first, the signal is divided into segments. Second, the rate is calculated as the sum of squares of the selected portion divided by the sum of squares of the entire signal. In our features list, the signal was split into ten pieces and the ratio was calculated for pieces one and eight.

Furthermore, we have the autocorrelation with lag = 2 (F10) meaning the correlation between values two samples apart. Besides, the $r_{sigma}=r\times std\left(x\right)$ with r=1.5 (F15) as the ratio of values that are $r_{sigma}$ away from the mean of the signal. A higher order autocovariance calculus is the $C3=\frac{1}{n-2lag}\sum_{i=0}^{n-2lag}x_{i+2\cdot lag}^{2}\cdot x_{i+lag}\cdot x_{i}$ equation (F11), where lag is the separation between samples and it is a measure of the non-linearity of the data [66].

As feature F14, we have the largest fixed point of dynamics. To understand the nature of complex systems, the field of stochastic modeling employs differential equations. Still, there are many theories to describe the dynamic. One of those is to consider the process as a Langevin process, governed by Equation (4) (for the first-order differential). A simple version, would be to consider the time series model as a function of the state variable *x* and time *t* by; $D^{\left(1\right)}\left(x\left(t\right)\right)$ (deterministic part of the dynamic), $\sqrt{D^{\left(2\right)}}=constant$ (stochastic force), and a Gaussian white noise factor (N)(mean=0,variance=1). The gathered data construct the Langevin equation without knowing the system dynamic. The terminology fixed-point refers to points where the drift coefficient is $D^{\left(1\right)}\cdot x_{fixedpoint}=0$, and its derivative is used for reducing the complexity in the analysis of the stability of the data; positive derivative, means stable fixed point and negative for an unstable point. Another simplification is applied when setting the $D^{\left(1\right)}\left(x\left(t\right)\right)$ as a polynomial whose coefficients come from the Friedrich procedure, to going deep on how the reconstruction is done, please refer to [67,68]. Our point is to explain the functionality of this dynamic modeling for classifying our data. In conclusion, the largest fixed point of dynamic (F14) is the maximum value of $x_{fixedpoint}$, where the drift coefficient is zero.

(4) $$\dot{x(t)}=D^{\left(1\right)}\cdot x(t)+\sqrt{D^{\left(2\right)}}\cdot N\left(0,1\right).$$

The final feature is mean-change quantiles (F16), which is a procedure where a range is limited by a qH (quantile maximum) and qL (quantile minimum). Subsequently, inside those boundaries, the mean of the absolute changes of the signal is computed. With qH = 1 and qL = 0, the mean-change is done over the entire signal.

In summary, we have extracted a total of 16 features per DMA pair (11 pairs, making it 176 features).

### 5.2. Classifier Selection

With the 176 total selected features, we proceeded to find the best classifier architecture to map them onto the facial actions. In [48], there is evidence that SVM (Support Vector Machine) is a good option in particular for avoiding overfitting. Others [69,70] have also achieved excellent results by using SVM with mechanomyography signals. In addition to the SVM option, we also decided to experiment with standard Matlab^®^.

We retained 33% as hold-out from the training set for classifier fine-tuning and started by looking at the default setting for KNN (K-nearest neighbors), SVM, and Ensemble-classifiers (Bootstrap Aggregation (Bagging) and Subspace) were tested. The best performing candidates were then fine-tuned through to obtain the optimal hyperparameters. The automatic performance metric was “accuracy” defined as $\frac{TP\;+\;TN}{TP\;+\;TN\;+\;FP\;+\;FN}$ where TN = True-negatives and FP = False-positives.

We used grid-search used for hyper-parameters improvement [71]. Grid-search is an exhaustive search based on a defined subset of the hyper-parameter space. In the SVM case, there exists a kernel parameter that we can use to estimate if our data are linearly or non-linearly separable. Besides, the reduction of the overfitting is tuned by the error penalty parameter (C). Using grid-search, we tested the kernel to 2 types, one linear and the other as a polynomial. We searched the best fit for a range of values of the regularization parameter (C) equal to [0.001,0.01,1,10], in case of polynomial, C = [7, 8, 9, 10, 12, 15, 20], degrees-options = [1, 2, 3] and the γ was set to [$1*4/n_{features}$, $1*16/n_{features}$, $1/n_{features}$, $1/4\cdot n_{features}$, $1/16\cdot n_{features}$], where $n_{features}=Top16_{Features}*DMA_{Combinations}$ and in the user-independent case the Gaussian kernel was added with C = [3, 5, 6, 7, 8, 9]. The validation of the grid-search selected was with 10fold cross-validation, and the performance metric was “recall” defined as $\frac{TP}{TP\;+\;FN}$; where, TP = True-positives and FN = False-negatives.

Accordingly, we focused on SVM in python using the scikit-learn library version 0.23.1 and compared the result with the standard Matlab classifiers as a baseline.

## 6. Evaluation Results

**Figure 11:**
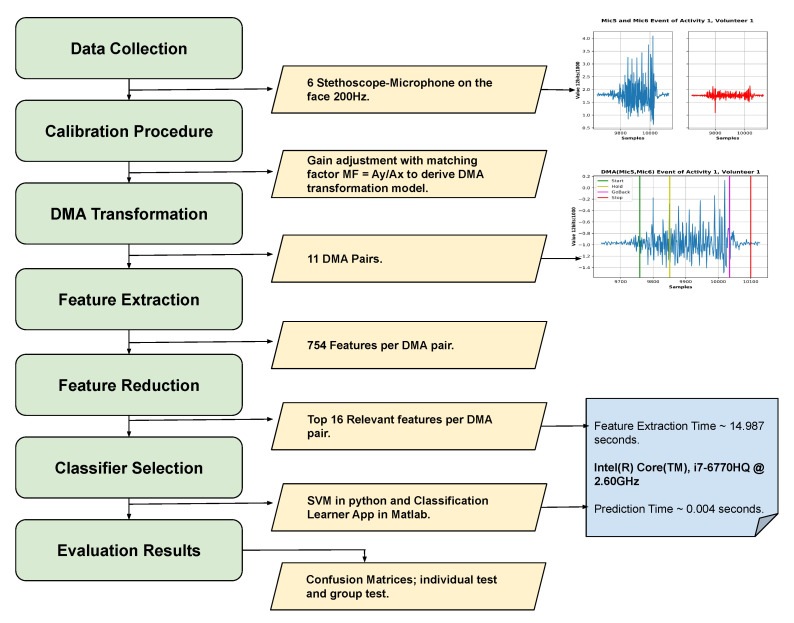
Flow diagram of the entire process done in this work.

The results described in this section are based on the processing chain shown in Figure 11. In this section, we present the results for both the user-independent and the user-dependent cases, including the differences between individual users and gestures and the influence of using different DMA pairs.

### 6.1. User-Dependent Test

**Figure 12:**
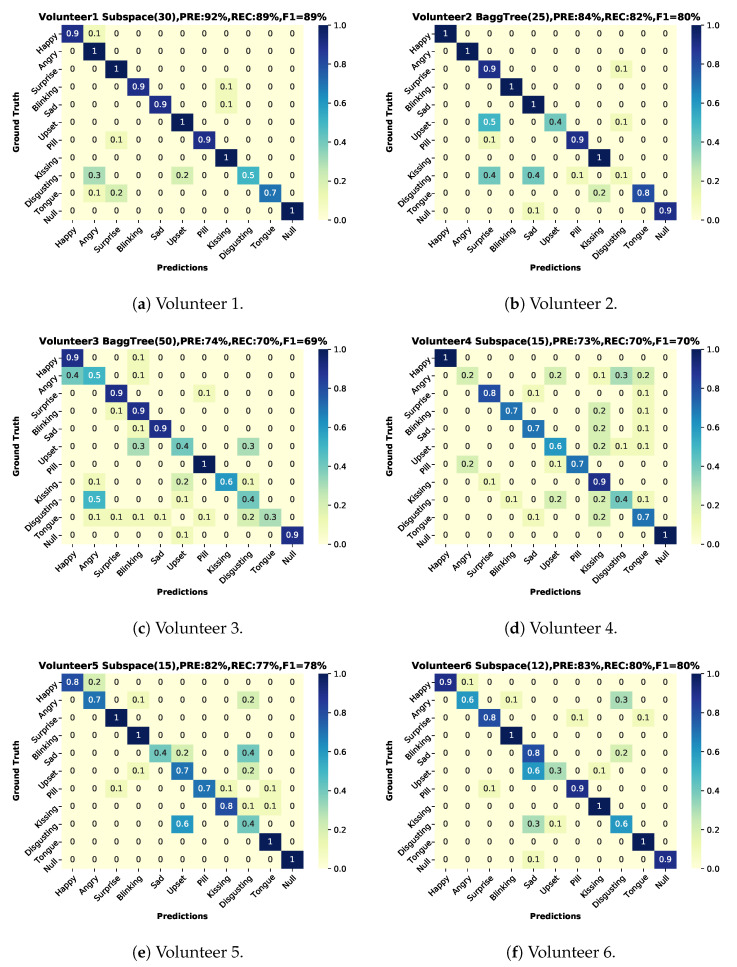

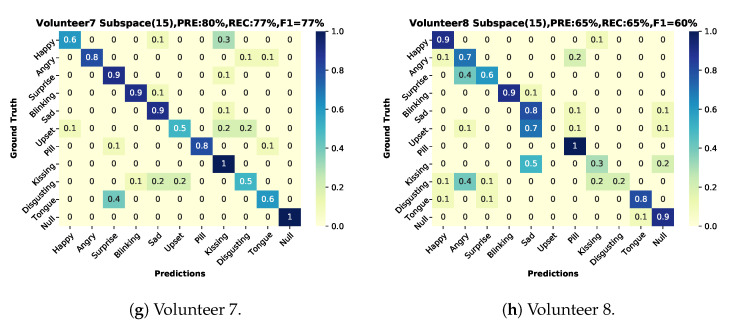
Confusion matrices user-dependent test, two sessions for training and one for testing with classifier, precision, recall and f1-score. (**a**) Volunteer-1 confusion matrix. (**b**) Volunteer-2 confusion matrix. (**c**) Volunteer-3 confusion matrix. (**d**) Volunteer-4 confusion matrix. (**e**) Volunteer-5 confusion matrix. (**f**) Volunteer-6 confusion matrix. (**g**) Volunteer-7 confusion matrix. (**h**) Volunteer-8 confusion matrix.

**Figure 13:**
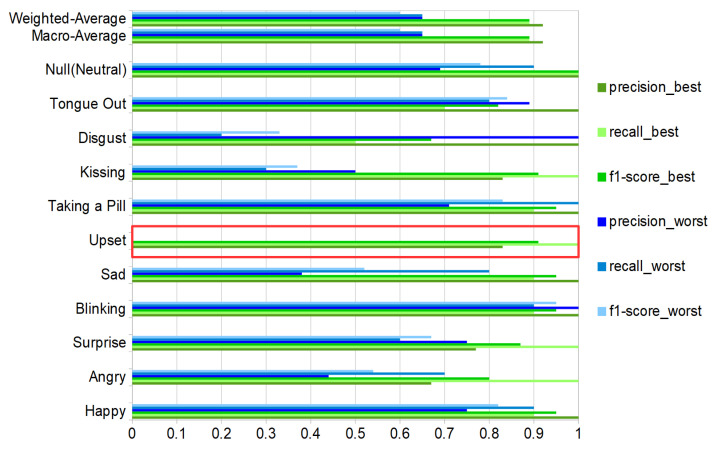
Best (green bars) and worst (blue bars) volunteer classification results for the ten activities and the null-class, with precision, recall and f1-score. The red box shows the upset expression with evaluation metrics equal to zero values for the worst case. Results with total test-events = 110 (10 per class, counting null-class).

The user-dependent results (training on two sessions and testing on the third one from the same user) are shown in Figure 12 based on a Matlab ensemble classifier with better performance than our python SVM implementation. The best result was volunteer 1(results on Figure 13, green color bars), for volunteers 1–7 we can see a more robust recall in comparison with volunteer number 8 (results on Figure 13, blue color bars).

All of these results were based on the selection of the maximum gain in “Area1”, which means that the matching factor is equal to $MF=A_{ymax}/A_{xmax}$. And it was selected by comparing the classification report for the gain = minimal, gain = maximum, gain = mean, and gain = 1 for the best volunteer. The results did not show any relevant differences. We selected the maximum due to a more balanced recall between classes for volunteer number 1, leaving out the recall for disgusting, which was the worst in all of the tested scenarios.

### 6.2. User-Independent Test

**Figure 14:**
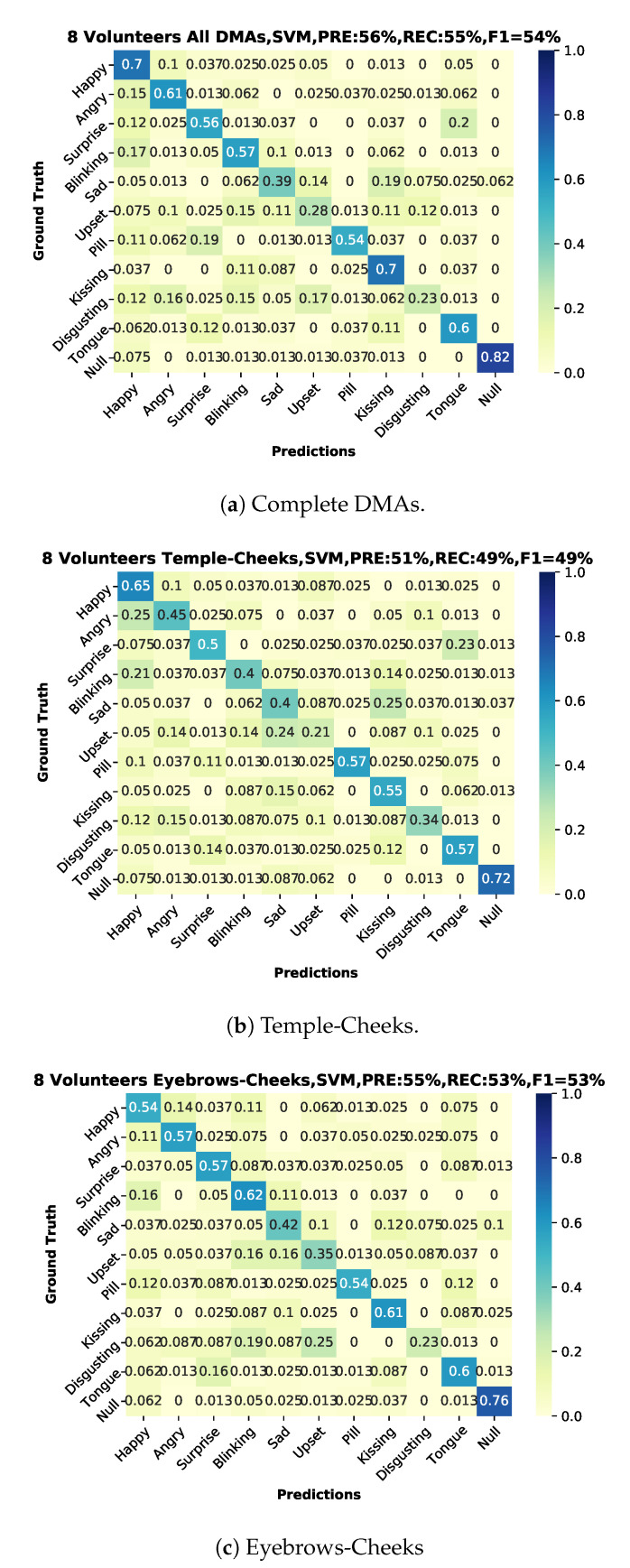
Confusion matrices user-independent, two sessions for training and one for testing with classifier, precision, recall and f1-score. (**a**) All volunteers using 11 combinations of first order DMAs. (**b**) All volunteers using 6 combinations of first order DMAs, only temples-cheeks. (**c**) All volunteers using 6 combinations of first order DMAs, only eyebrows-cheeks.

**Figure 15:**
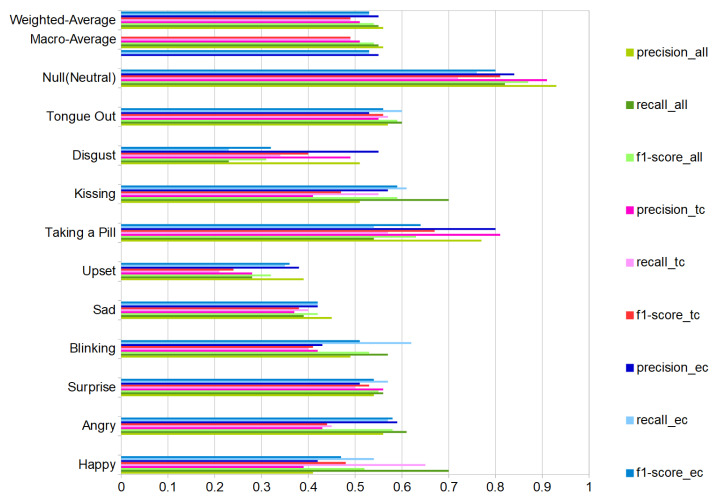
Classification results (Support Vector Machine) all volunteers for all DMAs (green bars), only temples-cheeks (red bars) and eyebrows-cheeks (blue bars). Ten activities and the null-class, with precision, recall and f1-score. Results with total test-events = 880 (80 per class, counting null-class).

We present the user-independent results in three confusion matrices corresponding to different selections of DMA pairs that were used for the recognition. One for the complete-case with all DMAs (11) in Figure 14a and two additional with a reduced number of DMA = 6; one just employing the stethoscope-microphones on the temple-cheeks in Figure 14b and a second one using the microphones on the eyebrows-cheeks presented in Figure 14c. We present an additional summary of the classification results in Figure 15 with green-tone color bars for 11 DMAs, as well as red-tone color bars and blue-tone color bars for the two 6 DMA cases. In all cases, the SVM (Gaussian kernel) classifier from scikit-learn was used, as it had the most consistent performance over all of the sensor configurations.

For the ten actions (+plus NULL class), we get an average F1-Score of 54%. This is not perfect, but significantly above chance, which confirms that our approach can extract relevant information about facial muscle activity patterns. In particular, 82% of NULL class means that we are reasonably good at picking relevant actions from noise.

We were not expecting a substantial difference between the temple-cheeks and eyebrows-cheeks setting, due to proximity of the stethoscope-microphone in the temple-eyebrows. We could consider this as a continuous sensor with four output points with a separation depending on the person’s forehead-temple distance. However, the results showed that the eyebrows-cheeks combination had the best-balanced recall inside our classes, besides a close relation with the complete case (all DMA). Therefore, we could conclude that the eyebrows-cheeks scheme is the most relevant. Moreover, as in the user-dependent experiment results, in the test with eight participants (user-independent), the emotions sad, upset, and disgusting are the weakest to recognize.

### 6.3. Discussion

#### 6.3.1. Overall Results

The best user-independent results are around 55% for precision, recall, and F1 score alike, as shown in Figure 14. This is far above a random result, which would be around 9%. This proves that (1) the differential sound signal from the chosen locations contains information about relevant facial actions and (2) our processing chain manages to extract much of this information. The fact that the error is not equally distributed, but instead some classes are recognized much better than others, is an indication that the results are not limited by system noise but by the actual information content (see Section 6.3.3).

The results must be seen in the context of two things that make achieving good results difficult and indicate that the approach is suitable for real-life applications. First, given the diversity and complexity of facial gestures, from the point of view of machine learning, the training data set is relatively small. Second, as described in Section 4.3, each user recorded three sessions with a long (hours or days) pause between sessions and, most importantly, the sensors being removed and placed again on the user before each session. This means that sensor placement inaccuracies/variations, which are major concerns in many wearable applications, are already factored in the results.

#### 6.3.2. User Dependence

Not surprisingly, as shown in Figures and Figure 13, user-dependent results: (1) are significantly better than the user independent ones and (2) show significant variations between users. Thus the lowest user-dependent F1 score is 60% (Volunteer 8) with is five percent more than the user-independent F1 while the score for the best user goes up to 89% (Volunteer 1). The differences between users can be attributed to three sources:

1. Physiological differences between users.
2. Different ways users may express specific actions.
3. Related to the above point, the inability of some subjects to mimic specific actions accurately.

A detailed understanding of which of the above accounts for which aspects of the system’s performance requires further research, including a more detailed analysis of the correspondence between physiological actions and the sound signals. Preliminary indications can be inferred from some qualitative observations. Thus, the most accurate volunteer was the person whose expressions were easier to decode by an observer. In the third volunteer case, we noticed that this person was doing exaggerated imitations compared to the rest and was commonly moving the entire face in all the gestures. In the case of volunteers 4 and 8, their movements were more subtle than the rest.

Overall, given the small amount of data (in terms of machine learning) and a small sample of users (8), we argue that the user-independent results are already quite promising. The next step must be to assemble a large number of users representative both in terms of physiology and the type of expressions, and investigate how advanced deep learning methods can generalize those for a more robust user-independent recognition.

#### 6.3.3. Gesture/Action Dependence

As expected, the recognition results vary sharply across the different gestures and actions. This is true for the user-independent case (70%F1 score for happy and kissing with all DMA vs. 28/23% for upset and disgust) and the user-dependent case (blinking with nearly identical F1 score around 90% for best and worst volunteer vs. upset where the best is 100% and the worst 0%), as illustrated in Figure 13.

In terms of most confusions, a few pairs of classes go up to 20–25% (depending on the sensor setup) in the user-independent case. As for example, surprise and sticking out the tongue (20%) is easily explained by the similarity between gestures (both have an open mouth, wide-open eyes), which results in both similar signals. For others, like taking a pill and surprise (19%), there is a little obvious similarity in the way they look, and the explanation must be in the externally non-visible muscle activation patterns (which need to be investigated further).

#### 6.3.4. Comparison with Other Published Approaches

In [12], a light sensor-based system option is presented with reliable results between 78.1–92.8% for the classification of six of the expressions in our facial gestures dictionary (neutral, happy, disgust, angry, surprise, fear, sad, and contempt). We only achieved such performance in the user-dependent case. However, as explained above, there is reason to believe that with a more extensive training set, the user-independent results should also significantly go up for our approach, in particular, if the training set can account for the natural variability of human facial expressions ([6]). The advantage of our design over the photo-light approach is robustness towards light condition, being more suitable for outside activities. Vision-based approaches, as in [1], also achieve high accuracies (>70 accuracy on average) however are, in general, non-wearable and involve privacy issues. A non-visual sensor solution like ours is an alternative option with only wearable settings. In the wearable domain, the most established approach is EMG with high accuracy for facial expression(smiling, neutral, and frowning), as in [14] (precision around 90%) or in [15] with an accuracy ≥90% for facial gestures, such as micro-smile, no-expression, smile, and laughter. As already explained in the introduction, the disadvantages of EMG are the age dependency (inversely proportional to the age), the person’s weight dependency, and the reduction in the discrimination of adjacent muscles [16]. Limitations that are not present in the stethoscope-microphone approach.

In Table 2, we have summarized key previously published approaches to non-vision based analysis of facial actions according to employed sensors, number of participants (average of 15.25), the total number of samples (average of 1885.25), number of experiment repetitions (average 2), placement of the sensors (typical glasses frame positions), set of expressions (average 6.8), and performance results (a direct comparison is not possible). The number of volunteers (20 with five repetitions) used in [17] is because, in this work, an additional cognitive-load experiment was designed (minimal 20 participants [72]), in our research, we are only evaluating the facial muscular movements with sound. It is imperative to highlight that our experimental design was never intended to be a psychology experiment, and it is a hardware sensing feasibility evaluation.

In Table 2, the results for the user-independent case are characterized by a reduction of performance around 50% when compared to the user-dependent case.

Our expression dictionary contains the highest number of facial gestures (11), which also reduces the chance level to 1/11=0.09 in comparison with our stronger competitor (Photo-reflective) with a chance level around 1/8=0.125 and with the half of our number of samples.

## 7. Conclusions and Future Work

We have demonstrated the feasibility of using differential sound mechanomyography as an unobtrusive mechanism for sensing facial muscle activity patterns. In particular, we have shown that sensors placed at locations corresponding roughly to the outline of typical smart glasses can provide enough information about muscle activity on the face as a whole to reliably identify meaningful expressions and face actions (f1-score of 54% as opposed 9% chance-level). Key specific takeaways are:

1. Using differential signals between suitable pairs of microphones is a key feature of our system. This is probably related to the fact that it captures temporal patterns of muscle activation rather than a precise sound corresponding to the specific type of activation of a particular muscle. It also helps us deal with inter person variability and noise.
2. The eyebrows-cheeks’ positions are the most informative locations for most of the investigated gestures and actions.
3. Using a stethoscope like sound acquisition setup has significantly improved the signal quality.
4. In our tests, we used a “train on all-test on all” approach, which demonstrates that the method has a degree of user independence. On the other hand, we have also seen a strong dependency on the person’s ability to recognize and mimic the expressions with the best user reaching an f1-score = 89% and the worst one being 60%.

We are currently working on a miniaturized version of the system truly integrated into a glasses frame in terms of future work. This will allow for us to go from mimicked expressions in a lab setting to recognizing real emotions under realistic circumstances. We will also investigate the fusion of differential sound information with other sensing modalities in particular with EMG, (simple) EEG, and our textile pressure sensor arrays based mechanomyography [17].

## Figures and Tables

**Table 1:** A=V·G/1000 per Microphone in Area 1.

Mic_1	Mic_2	Mic_3	Mic_4	Mic_5	Mic_6
min = 2.372	min = 1.486	min = 1.217	min = 1.108	min = 1.071	min = 1.015
max = 3.219	max = 2.254	max = 2.122	max = 2.737	max = 4.095	max = 1.811
mean = 2.787	mean = 1.953	mean = 1.701	mean = 2.103	mean = 2.656	mean = 1.451

**Table 2:** Comparison with state-of-the-art non-visual methods for facial expressions recognition. Human Participant Pool (HPP). Repetitions of entire experiment (REP).

Study	Description	Participants, Experiment Repetitions and Samples(HPP-REP)	Location	Expressions	Performance
Our Approach	Sthetoscope DMAs.	8-3 with time gap. [2400 + 240 (Neutral)] Samples.	Eyebrows (LOC1), Cheeks (LOC2) and Temples (LOC3).	Happiness, anger, surprise, sadness, upset and disgusted, and gestures as kissing, winkling, sticking the tongue out, taking a pill and neutral. Total = 11.	** User-dependent = 60–89% f1. * User-independent (LOC1 and LOC2) = 53% f1, ** User-independent (LOC2 and LOC3) = 49% f1.
Photo Reflective [12]	17 Photo sensors.	** Case A: 8-1 without time gap. 960 Samples (8 expressions X 15 poses per volunteer). * Case B: 3-3 different days. 24 Samples (8 expression X 1 pose per volunteer).	Glasses Frame.	Neutral, happy, disgust, angry, surprise, fear, sad, contempt. Total = 8.	** Case A: User-dependent = 84.8–99.2% accuracy. (50% Training). * User-independent with leave-volunteer out = 48% accuracy. ** Case B: User-dependent with leave-session out (3 volunteers) = 78.1% accuracy.
TPM [17]	Textile Pressure Sensors.	20-5 with time gap. 6000 Samples.	Forehead.	Joy, surprise, sadness, neutral, fear, disgust, anger. Total = 7.	** User-independent = 38% accuracy (Five-fold cross validation).
EMG Gruebler [14]	3 Electrode pairs.	10-1. 160 Samples (4 repetitions for each expressions).	Temples.	Neutral, smiling, frowning, and neither (biting and neutral). Total = 4	** User-dependent => 80% accuracy for Smiling and Frowning (training = 3 repetitions per expression)
EMG Perusquia [15]	Four surface EMG channels.	23-1. 238 smiles, 32 micro-smiles and total of 421 expressions.	Temples.	Micro-smile, no-expression, smile, and laughter. Total = 4	** User-dependent => 90% accuracy.
